# Idiopathic Fascicular Ventricular Tachycardia

**Published:** 2004-07-01

**Authors:** Johnson Francis, Venugopal K, Khadar S A, Sudhayakumar N, Anoop K Gupta

**Affiliations:** * Department of Cardiology, Medical College Calicut, Kerala, India; † Department of Cardiology, Medical College Kottayam, Kerala, India; ‡ Apollo Hospital, Ahmedabad, India

**Keywords:** Ventricular Tachycardia, Structural Normal Heart, Structural Normal Heart

## Abstract

Idiopathic fascicular ventricular tachycardia is an important cardiac arrhythmia with specific electrocardiographic features and therapeutic options. It is characterized by relatively narrow QRS complex and right bundle branch block pattern. The QRS axis depends on which fascicle is involved in the re-entry. Left axis deviation is noted with left posterior fascicular tachycardia and right axis deviation with left anterior fascicular tachycardia. A left septal fascicular tachycardia with normal axis has also been described. Fascicular tachycardia is usually seen in individuals without structural heart disease. Response to verapamil is an important feature of fascicular tachycardia.  Rare instances of termination with intravenous adenosine have also been noted. A presystolic or diastolic potential preceding the QRS, presumed to originate from the Purkinje fibers can be recorded during sinus rhythm and ventricular tachycardia in many patients with fascicular tachycardia. This potential (P potential) has been used as a guide to catheter ablation. Prompt recognition of fascicular tachycardia especially in the emergency department is very important. It is one of the eminently ablatable ventricular tachycardias. Primary ablation has been reported to have a higher success, lesser procedure time and fluoroscopy time.

## Introduction

In general ventricular tachycardias have wide QRS complexes.  One of the earliest descriptions of ventricular tachycardia (VT) with a narrow QRS complex was by Cohen et al in 1972 [1]. Their description was a left posterior fascicular tachycardia with relatively narrow QRS. In 1979, Zipes et al [2] reported three patients with ventricular tachycardia characterized by QRS width of 120 to 140 ms, right bundle branch block morphology and left-axis deviation. These patients were young and had no major cardiac abnormalities. The arrhythmia could be induced by exercise, atrial and ventricular premature beats as well as atrial pacing and ventricular pacing. Belhassen et al observed that this tachycardia can be terminated by the calcium channel blocker verapamil [3] This observation has been confirmed subsequently by others as wells [4-7]. Belhassen et al proposed that this is a specific ECG-electrophysiological entity [8]. Fascicular tachycardia has also been called Idiopathic Left Ventricular Tachycardia (ILVT) by other authors, though left ventricular outflow tract VT also comes under the purview of this term [9,10]. Fascicular tachycardia is usually paroxysmal, but a case which was persistent, leading to cardiac enlargement and complete resolution following therapy with verapamil has also been reported [4]. Termination of idiopathic fascicular ventricular tachycardia by vagal maneuvers was noted in 4 cases by Buja  et al. [11]. Successful radiofrequency catheter ablation was described by Klein et al. [12]. In this article we propose to review the current status of our knowledge regarding the genesis and treatment of idiopathic fascicular ventricular tachycardia.

## Mechanism and Classification

Zipes et al postulated that the origin of the tachycardia was localized to a small region of reentry or triggered automaticity located in the posteroinferior left ventricle, close to the posterior fascicle of the left bundle branch.2Response to verapamil suggested a role for the slow inward calcium channel in the genesis of the arrhythmia. Endocardial mapping during tachycardia revealed the earliest activation at the ventricular apex and mid septum [13]. The tachycardia can be entrained by ventricular and atrial pacing. Entrainment by atrial pacing suggests easy access over the conduction system into the reentry circuit and hence a role for the fascicles in the reentrant circuit [14]. Lau suggested the origin as reentry circuits involving the lower septum or posterior part of the left ventricle close to  the endocardial surface in view of the response to radiofrequency ablation in these sites [15]. Purkinje potential recorded in the diastolic phase during VT at the mid-anterior left ventricular septum in rare cases with RBBB pattern and right axis deviation suggested origin near left anterior fascicle in those cases [16].

Recently Kuo et al has questioned the involvement of the fascicle of the left bundle branch in ILVT [17]. They studied two groups of patients with ILVT. One with left anterior or posterior fascicular block during sinus rhythm and the other without. They noted that the transition zone of QRS complexes in the precordial leads were similar during VT in both groups. New fascicular blocks did not appear after ablation. Therefore they concluded that  the fascicle of the left bundle branch may not be involved in the anterograde limb of reentrant circuit in ILVT. 

**Figure 1:**
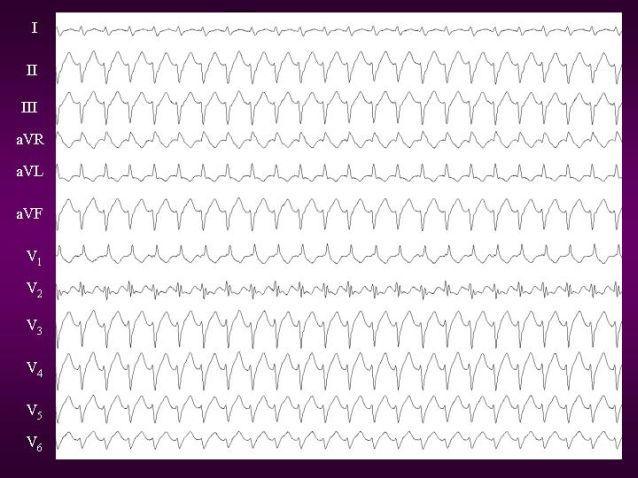
12 lead ECG of Idiopathic left ventricular tachycardia. It shows classical RBBB with leftward axis morphology suggestive of posterior fascicle origin.

Fascicular tachycardia has been classified into three subtypes: (1) left posterior fascicular VT (Figure 1) with a right bundle branch block (RBBB) pattern and left axis deviation (common form); (2) left anterior fascicular VT with RBBB pattern and right-axis deviation (uncommon form); and (3) upper septal fascicular VT with a narrow QRS and normal axis configuration (rare form) [18].

## Anatomical Substrate

Endocardial activation mapping during VT identifies the earliest site in the region of the infero-posterior left ventricular septum. This finding, along with VT morphology and short retrograde VH interval suggests a left posterior fascicular origin. Nakagawa and colleagues [19] recorded high-frequency potentials preceding the site of earliest ventricular activation during the VT and sinus rhythm. These potentials are thought to represent activation of Purkinje fibers and are recorded from the posterior one third of the left ventricular septum. Successful RF ablation is achieved at sites where the purkinje potential is recorded 30 to 40 ms before the VT QRS complex.

Some date suggest that the tachycardia may originate from a false tendon or fibro- muscular band that extends from the posteroinferior left ventricle to the basal septum [20]. Histological examination of false tendon disclosed abundant Purkinje fibers.

## Electrophysiological Study 

Fascicular tachycardia can be induced by programmed atrial or ventricular stimulation in most cases. Isoprenaline infusion may be required in certain cases; rarely there may be difficulty in induction despite isoprenaline infusion. Endocardial mapping identifies the earliest activation in the posteroapical left ventricular septum in patients with posterior fascicular tachycardia.

**Figure 2:**
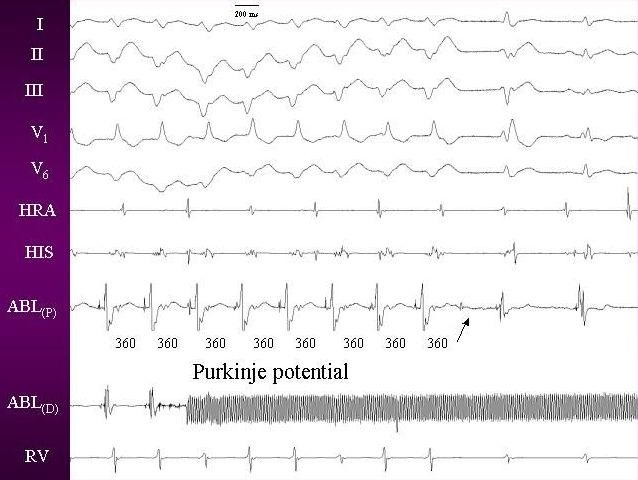
Intracardia electrogram during tachycardia showing purkinje potential, which persisted after the ablation also (arrow).

A high frequency potential with short duration, preceding the QRS has been described as the Purkinje potential (Figure 2). This has also been called P potential and diastolic potential. P potentials can be recorded both in sinus rhythm and during ventricular tachycardia. Pacing at sites manifesting the earliest P potential produces QRS complexes identical to that of the clinical tachycardia [19].

## Pharmacological Therapy

Intravenous verapamil is effective in terminating the tachycardia. However the efficacy of oral verapamil in preventing tachycardia relapse is variable. Good response and resolution of tachycardiomyopathy with verapamil treatment was noted by Toivonen et al [4], while Chiaranda et al commented on the poor efficacy [21]. Treatment with propranolol has also resulted in cure of arrhythmia and resolution of features of tachycardiomyopathy in another case with incessant fascicular VT [221]. Though fascicular tachycardias do not generally respond to adenosine, termination of VT originating from the left anterior fascicle by intravenous adenosine has been documented [23].

## Catheter Ablation

The young age of most patients with need for long-term antiarrhythmic treatment and attendant side effects prompted the search for curative therapies. Fontaine et al (1987) described successful treatment of ILVT by application of a high-energy DC shock (fulguration) between the catheter tip and a neutral plate placed under the patient's back [24]. Klein et al (1992) reported cure of ILVT by radiofrequency catheter ablation [25]. Since then radiofrequency has remained the procedure of choice.

Different approaches for radiofrequency ablation have been described by various authors. Nakagawa et al preferred careful localization of the Purkinje potential in guiding ablation. They selected the area where a Purkinje potential precedes the QRS complex during tachycardia [19]. Wellens et recommend pace mapping with a match between the 12 simultaneously recorded ECG leads during pacing and the clinical tachycardia for localizing the site of ablation [9]. They hypothesize that pathways within the Purkinje network that are not included in the reentry circuit responsible for the tachycardia may also become activated. Ablation of those regions may not result in interruption of the tachycardia circuit.

## Primary Radiofrequency Ablation

Since fascicular VT is sometimes difficult to induce despite pharmacological provocation, some workers (Gupta et al) prefer primary ablation. In a recent report, seven cases of incessant fascicular VT were successfully ablated with no recurrence [26]. They reported a shorter procedure time, significantly lower fluoroscopy time and lesser number of radiofrequency energy deliveries in the primary versus elective groups. The longer procedural time during elective ablation was mainly due to the time spent in induction of  fascicular VT.
